# Application of the precision eye health education model in myopia prevention and control in adolescents

**DOI:** 10.3389/fped.2025.1554822

**Published:** 2025-05-08

**Authors:** Qi Wang, Lili Zhang, Yanmei Wu

**Affiliations:** ^1^ Department of Ophthalmology, Panjin LiaoHe Oil Field Gem Flower Hospital, Panjin, Liaoning, China; ^2^ Department of Research, Panjin LiaoHe Oil Field Gem Flower Hospital, Panjin, Liaoning, China; ^3^ Department of Dean's Office, Panjin LiaoHe Oil Field Gem Flower Hospital, Panjin, Liaoning, China

**Keywords:** myopia, adolescents, eye health education, precision eye health education model, questionnaire on knowledge, attitude, practice, satisfaction rate

## Abstract

**Objective:**

This study aimed to evaluate the application value and feasibility of a precise eye health education model in the prevention and control of myopia in adolescents.

**Methods:**

Adolescent students were assigned to either an experimental group or a control group, with both groups comprising one class from each of the seventh, eighth, and ninth grades across three schools. The experimental group received interventions based on the precision eye health education model, whereas the control group underwent a conventional health education program. One month after the intervention, assessments were conducted to evaluate students' eye health knowledge, attitudes, and practices (KAP), as well as their level of satisfaction with the format and content of the eye health education.

**Results:**

A total of 461 students were included in the experimental group, while 443 students were in the control group. One month post-intervention, the experimental group demonstrated significantly higher scores in eye health knowledge, attitudes, and practices than the control group (*P* < 0.05). Additionally, students in the experimental group reported greater satisfaction with both the format and content of the educational intervention than those in the control group (*P* < 0.05).

**Conclusion:**

The application of a precision eye health education model for myopia prevention and control in adolescents effectively enhances students' knowledge, attitudes, and practices regarding eye health. Additionally, this model is associated with a high level of student satisfaction, highlighting its potential as a more effective approach to adolescent eye health education.

## Introduction

Myopia, often known as short-sightedness or near-sightedness, is a prevalent condition that typically develops during childhood (1), posing long-term risks to visual health and overall well-being. Beyond impairing daily quality of life, myopia can lead to severe ocular complications, including retinal detachment, retinal neovascularization, early-onset cataracts, and glaucoma (2, 3). Globally, myopia has become an urgent public health concern, particularly in East Asia, where its incidence has risen sharply, although prevalence rates vary across different countries (1). China, where this study is conducted, faces a similarly severe myopia epidemic, presenting a substantial public health challenge.

China has introduced the Comprehensive Plan to Prevent Nearsightedness among Children and Teenagers. Key strategies outlined in the plan include promoting outdoor activities and physical exercise, optimizing light exposure, regulating near-work habits, limiting screen time, integrating traditional Chinese eye exercises, maintaining a balanced diet, and ensuring adequate sleep (4). As a crucial component of these efforts, traditional eye health education seeks to enhance public awareness and promote preventive practices through the dissemination of eye care knowledge. However, its practical effectiveness remains limited, often constrained by parents' understanding, acceptance, and implementation of recommended interventions (5). This highlights the need for more precise and targeted educational approaches to effectively address the growing myopia crisis.

In recent years, the limitations of traditional eye health education have prompted growing interest in precision eye health education. Precision health, an emerging trend in modern healthcare, builds upon the principles of precision medicine but shifts the focus from treating diseases to proactive health management and disease prevention (6). While precision medicine primarily aims to diagnose and treat conditions once they arise, precision health emphasizes preventive strategies and personalized interventions, empowering individuals to take charge of their well-being. This approach involves assessing biological, genetic, social, economic, cultural, and environmental factors to develop tailored healthcare solutions, preventive measures, and health-promoting strategies. By leveraging disease prediction models, precision health seeks to enable individuals to maintain optimal health and reduce disease risk (6). To mitigate the potential risks associated with myopia, precision education emphasizes a customized health education model that transcends traditional methods by integrating the collaborative efforts of schools, families, and society, and leveraging modern technological innovations such as artificial intelligence. It focuses on enhancing public education in family and school settings and raising parental awareness (7). Moreover, the rising prevalence of myopia has been linked to high-intensity, high-pressure educational environments, further underscoring the urgency of adapting social practices to alleviate this issue (8). The rapid advancement of artificial intelligence and digital technologies, such as telemedicine, has shown great potential in addressing global healthcare challenges, including myopia prevention and control (9). Numerous studies have already explored the integration of AI and digital technology into myopia management, with some yielding remarkable outcomes (10).

By harnessing the strengths of precision health, school-based interventions, family involvement, medical expertise, and digital technologies, a precision eye health education model presents a more effective and sustainable approach to combating myopia in adolescents. Based on this framework, this study aims to assess the feasibility and effectiveness of a precision eye health education model in myopia prevention and control among adolescents, offering a forward-thinking solution to a growing public health concern.

## Materials and methods

### Baseline information

In September 2024, a randomized sampling method was employed to select a public middle school with comparable educational backgrounds from three districts: Xigang District in Dalian, Linghe District in Jinzhou, and Xinglongtai District in Panjin. From each selected school, two classes from Grades Seven, Eight, and Nine were randomly chosen to participate in the study. Subsequently, the classes within each grade across the three schools were stratified using a random odd-even numbering approach, resulting in the formation of an experimental group and a control group. Each group consisted of one class from each of the three grades in the respective schools.

### Inclusion criteria

The study applied the following inclusion criteria: (1) Students aged 12–16 years from the selected schools in the three districts, all with comparable educational backgrounds; (2) Students in good health, able to participate in daily school activities and academic programs; (3) Students continuously enrolled in the designated classes with no prior plans for transfer and no actual transfer occurring during the study period; (4) Students willing and able to cooperate with the intervention measures, relevant assessments, and questionnaires; (5) Students and their guardians who provided informed consent to participate in the study; (6) Ethical approval obtained for the study.

### Exclusion criteria

Students meeting any of the following criteria were excluded from the study: (1) Students who had participated in other eye health education programs or therapeutic studies within the past 3 months; (2) Students with conditions or disabilities that significantly impaired normal learning and daily activities, such as severe psychological disorders or major illnesses; (3) Students unable to complete the interventions or assessments due to various reasons, such as anticipated transfer or prolonged absence; (4) Students diagnosed with severe eye diseases, including but not limited to nystagmus, glaucoma, cataracts, photosensitivity, or retinal detachment; (5) Students with incomplete or missing baseline data or questionnaire information.

### Methods

#### Control group

The control group received a conventional eye health education model, which encompassed standard education and guidance provided by schools, society, and the Center of Disease Control (CDC). This model involved disseminating knowledge on myopia prevention and control through various approaches, such as organizing centralized lectures, distributing informational handbooks, and creating educational display boards. Additionally, students and their families received prompt and proactive responses to their questions regarding adolescent myopia. This education was conducted once per month.

#### Experimental group

The experimental group received a precision eye health education model, which incorporated targeted interventions in addition to the conventional eye health education model. The interventions included the following components:

(1) A multidisciplinary health education team was established, comprising representatives from medical institutions, the CDC, schools, and technology platform technicians. The responsibilities of each entity were as follows: ① The CDC provided scientific guidance for the overall health education framework. ② Medical institutions developed educational content on myopia prevention and disseminated it to students and parents via an intelligent eye health management cloud platform. ③ School health teachers and class teachers, after undergoing training by the project team, were responsible for delivering and supervising offline health education content. ④ Technology personnel managed the cloud platform's daily operations and maintained its backend functions.
(2) The eye health intelligent management cloud platform was leveraged for intelligent content delivery. This content included online health education, vision monitoring and alerts, comprehensive intervention strategies, and dynamic management of eye health data. Students and parents could access the platform in two ways: by scanning a QR code linked to student screening information, or by inputting identity credentials through an official account or mini-program. This enabled real-time updates on personalized eye health knowledge, continuous tracking of eye health data, and adaptive management of intervention measures.
(3) Using baseline data from a prior project, students were categorized into three groups: general, high-risk, and affected groups. The health education content was customized for each group. This content included universal, personalized, interactive, and integrated online-offline approaches to myopia prevention and control.
① Universal education encompassed key topics such as healthy vision concepts, behaviors related to eye use, dietary habits, sleep hygiene, optimal eye environment, and relevant policies and regulations. The cloud platform helped spread knowledge. It did this by sending out 2–3 popular science articles each week. This allowed students to learn flexibly, without being limited by time or space. This systematic method slowly built awareness about eye health. It also increased how often students were exposed to information. This promoted a more effective and precise way to prevent myopia in adolescents.
② Personalized education was supported by an established myopia prevention and control system. Throughout different stages, such as screening, prevention, diagnosis, treatment, and management, the cloud platform dynamically tracked eye health data and monitored intervention measures. Participants recorded their daily eye usage habits on the platform. The platform then automatically generated weekly reports to help them clearly see their behavioral changes. Based on individual needs, targeted education was offered. This included behavior modification, myopia treatment guidance, and prevention of potential complications.
③ Interactive education focused on vision monitoring data. Regular data aggregation and analysis were performed through the platform's dynamic management system. This analysis enabled real-time sharing of students' vision and behavioral patterns with parents and students. Parents received automated reminders regarding their child's vision progress. Also, an online consultation system was set up to handle any questions.
④ For integrated online and offline health education, a balanced allocation of resources was achieved by seamlessly combining digital and in-person educational strategies. Online, health education content was spread through platforms. At the same time, regular seminars and training sessions were conducted for health education personnel from the CDC, medical institutions, and schools. The aim was to improve their knowledge of eye health education. Building on this training, schools added age-suitable eye health education to different offline activities. These included sports classes, class meetings, and extracurricular programs. To further reinforce healthy vision habits, weekly outdoor activity sessions were organized. Moreover, a variety of interactive and engaging formats were used. These formats included student-led discussions, peer self-assessments, handwritten health bulletins, themed class meetings, and special projects. They were employed to spark students’ enthusiasm for maintaining healthy lifestyles. This multi-faceted approach fostered active participation, strengthened health awareness, and encouraged long-term adherence to myopia prevention and control strategies.

### Observation indicators

(1) The eye health knowledge, attitude, and practice (KAP) questionnaire: A self-designed KAP questionnaire was employed to assess participants' knowledge, attitudes, and practices regarding eye health. The questionnaire consisted of three dimensions, namely eye health knowledge, attitudes towards eye health, and eye health practices. The reliability and validity of the questionnaire were confirmed, with a test-retest reliability coefficient of 0.854, Cronbach's alpha coefficient of 0.718, and split-half reliability coefficient of 0.765, indicating good internal consistency and stability. The calculation formula and scoring criteria were as follows: eye health knowledge consisted of 16 true-or-false questions, with one point awarded for each correct response and zero points for incorrect responses. The total possible score was 16 points, with higher scores indicating a stronger awareness of eye health knowledge. Attitudes towards eye health included 10 multiple-choice questions, scored on a scale of 4, 3, 2, and 1 according to different levels of positive attitudes towards eye health. The total possible score was 40 points, with higher scores reflecting a more positive attitude. Eye health practices consisted of 12 multiple-choice questions assessing weekly behavioral habits. Responses were rated on a 3, 2, and 1 scale, with a total possible score of 36 points. A higher score represented better eye health behavior habits. Assessments were conducted 1 month after the intervention. All participating adolescents completed the questionnaire independently under the supervision and guidance of their class teachers and trained surveyors. The distribution and collection of questionnaires were handled by surveyors who had undergone standardized training to ensure consistency and data accuracy.
(2) Satisfaction with eye health education: One month after the intervention, a self-designed satisfaction questionnaire was administered, including two dimensions, namely the form and content of the health education. The questionnaire consisted of five questions, each rated on a 5-point Likert scale (scores ranging from 5 to 1). The total possible score was 25 points, with higher scores indicating greater satisfaction with the intervention.

### Statistical methods

Statistical analysis was performed using SPSS 26.0. Qualitative data were described as [*n* (%)] for *χ*^2^ tests. Skewed quantitative data were described as *M* (*p*25, *p*75) for Mann–Whitney *U* tests. When a difference equaling to *P* < 0.05 occurred, it was considered statistically significant.

## Results

### Questionnaire recovery status of both groups

A total of 916 KAP questionnaires on adolescent eye health were distributed, with 904 valid responses, yielding an effective response rate of 98.69%. In the experimental group, 464 questionnaires on KAP regarding adolescent eye health were distributed, with 461 valid responses, resulting in an effective response rate of 99.35%. In the control group, 452 KAP questionnaires were distributed, and 443 valid responses were received, achieving an effective response rate of 98.01%. All satisfaction questionnaires regarding the eye health education intervention were fully recovered, with a 100% recovery rate. Following statistical analysis, a total of 461 students from the experimental group and 443 students from the control group were ultimately included in the study. Baseline data analysis revealed no statistically significant differences between the two groups (*P* > 0.05), ensuring their comparability for the study (Table 1).

**Table 1:** Comparison of baseline information between the two groups [*n* (%)].

Indicator	Experimental group (*n* = 461)	Control group (*n* = 443)	*χ* ^2^	*P*
Gender (*n*)			0.030	0.864
Male	244 (52.93)	237 (53.50)	-	-
Female	217 (47.07)	206 (46.50)	-	-
Age			0.978	0.913
12 years old	132 (28.63)	132 (29.80)	-	-
13 years old	142 (30.80)	124 (27.99)	-	-
14 years old	123 (26.68)	120 (27.09)	-	-
15 years old	53 (11.50)	55 (12.42)	-	-
16 years old	11 (2.39)	12 (2.71)	-	-
Region			0.140	0.932
Xigang District	157 (34.06)	147 (33.18)	-	-
Linghe District	153 (33.19)	152 (34.31)	-	-
Xinglongtai District	151 (32.75)	144 (32.51)	-	-
Grade			0.016	0.992
Grade Seven	161 (34.92)	154 (34.76)	-	-
Grade Eight	154 (33.41)	147 (33.18)	-	-
Grade Nine	146 (31.67)	142 (32.05)	-	-
Myopia			0.570	0.450
No	133 (28.85)	138 (31.15)	-	-
Yes	328 (71.15)	305 (68.85)	-	-

### Comparison of eye health knowledge between the two groups

One month after the intervention, there were statistically significant differences (*P* < 0.05) in eye health knowledge scores between the experimental and control groups. Specific differences included knowledge of the following: the normal vision standard (≥5.0); the impact of reading, writing, or using electronic devices while lying down on vision; the effects of using electronic devices in a moving vehicle on vision; the consequences of reading, writing, or using electronic devices while walking; the impact of watching TV at night with the lights off on vision; the negative effects of picky eating and dietary imbalances on vision. Furthermore, students in the experimental group achieved higher total scores in eye health knowledge assessment compared to the control group (*P* < 0.05) (Table 2).

**Table 2:** Comparison of knowledge levels about eye health between the two groups (points) [*M* (*p*25, *p*75)].

Knowledge	Experimental group (*n* = 461)	Control group (*n* = 443)	*Z*	*P*
1. The vision of common people is generally ≥5.0	1.00 (1.00, 1.00)	0.00 (0.00, 1.00)	−17.781	<0.001
2. Myopia is characterized by the inability to see things clearly in the distance	1.00 (1.00, 1.00)	1.00 (1.00, 1.00)	0.000	1.000
3. Myopic people should insist on wearing glasses	1.00 (1.00, 1.00)	1.00 (1.00, 1.00)	0.000	1.000
4. Myopia is related to excessive eye use	1.00 (1.00, 1.00)	1.00 (1.00, 1.00)	0.000	1.000
5. Rest eyes after 30 min of continuous use	1.00 (1.00, 1.00)	1.00 (1.00, 1.00)	0.000	1.000
6. Vision loss should be examined and treated at the hospital in time	1.00 (1.00, 1.00)	1.00 (1.00, 1.00)	0.000	1.000
7. Reading and writing while lying down or using electronic products can easily lead to eye fatigue	1.00 (1.00, 1.00)	1.00 (1.00, 1.00)	0.000	1.000
8. Reading and writing while lying down or using electronic products affects vision	1.00 (1.00, 1.00)	1.00 (1.00, 1.00)	−2.506	0.012
9. Reading, writing or using electronic products in a moving car affects vision	1.00 (1.00, 1.00)	1.00 (1.00, 1.00)	−3.703	<0.001
10. Reading, writing or using electronic products while walking affects vision	1.00 (1.00, 1.00)	1.00 (1.00, 1.00)	−4.244	<0.001
11. Reading, writing or using electronic products in direct sunlight or under strong light affects vision	1.00 (1.00, 1.00)	1.00 (1.00, 1.00)	−1.020	0.308
12. Reading, writing or using electronic products in dimly lit places affects vision	1.00 (1.00, 1.00)	1.00 (1.00, 1.00)	0.000	1.000
13. Watching TV at night with the lights off affects vision	1.00 (1.00, 1.00)	1.00 (1.00, 1.00)	−5.379	<0.001
14. Picky eating and partiality of food are harmful to vision	1.00 (1.00, 1.00)	1.00 (1.00, 1.00)	−5.689	<0.001
15. Strengthening physical exercise is conducive to protecting vision	1.00 (1.00, 1.00)	1.00 (1.00, 1.00)	−5.595	<0.001
16. Doing eye exercises is beneficial to protecting vision	1.00 (1.00, 1.00)	1.00 (1.00, 1.00)	0.000	1.000
Total score for knowledge of eye health	16.00 (16.00, 16.00)	14.00 (15.00, 15.00)	−19.129	<0.001

### Comparison of attitudes towards eye health between the two groups

One month post-intervention, there were statistically significant differences (*P* < 0.05) in attitudes towards eye health between the experimental and control groups. Key differences were noted in the following statements: poor vision negatively impacts overall health; regular vision checkups should be conducted; good vision is more important than academic performance; acquiring proper knowledge and attitudes toward vision protection significantly contributes to maintaining good eyesight; willingness to improve dietary habits to protect vision; willingness to adjust bad eye habits while studying. Meanwhile, the experimental group exhibited a higher total score for attitudes towards eye health than the control group (*P* < 0.05) (Table 3).

**Table 3:** Comparison of attitude towards eye health between the two groups (points) [*M* (*p*25, *p*75)].

Attitude	Experimental group (*n* = 461)	Control group (*n* = 443)	*Z*	*P*
1. Poor vision affects health	4.00 (4.00, 4.00)	4.00 (4.00, 4.00)	−6.029	<0.001
2. Regular vision examinations should be conducted	4.00 (4.00, 4.00)	3.00 (3.00, 4.00)	−13.105	<0.001
3. Families and schools should pay attention to knowledge and measures to prevent myopia	4.00 (4.00, 4.00)	4.00 (4.00, 4.00)	0.000	1.000
4. Good vision is more important than good grades	3.00 (2.00, 3.00)	2.00 (2.00, 2.00)	−14.982	<0.001
5. Good knowledge of vision protection and correct attitudes can help a lot in vision protection.	4.00 (4.00, 4.00)	4.00 (4.00, 4.00)	−5.069	<0.001
6. Be willing to take part in physical exercise to protect vision	4.00 (4.00, 4.00)	4.00 (4.00, 4.00)	−1.723	0.085
7. Be willing to adjust bad dietary habits to protect vision	3.00 (4.00, 4.00)	4.00 (4.00, 4.00)	−2.847	0.004
8. Be willing to publicize the knowledge of myopia prevention to people around	3.00 (4.00, 4.00)	4.00 (4.00, 4.00)	−0.091	0.927
9. Be willing to change bad eye habits when studying	4.00 (4.00, 4.00)	4.00 (4.00, 4.00)	−3.726	<0.001
10. Be willing to wear corrective glasses for myopia	4.00 (4.00, 4.00)	4.00 (4.00, 4.00)	−1.299	0.194
Total score for the attitude towards eye health	37.00 (36.00, 38.00)	36.00 (35.00, 37.00)	−12.727	<0.001

### Comparison of eye health behaviors between the two groups

One month after the intervention, significant differences (*P* < 0.05) in eye health behaviors were observed between the experimental and control groups. The experimental group demonstrated better eye health habits in the following areas: avoiding reading, writing, or using electronic devices while lying face down; avoiding reading, writing, or using electronic devices while lying on the back or side; avoiding using electronic devices while in a moving car; avoiding using electronic devices while walking; avoiding reading, writing, or using electronic devices under direct sunlight or strong light exposure; avoiding reading, writing, or using electronic devices in dimly lit environments; avoiding watching TV at night with the lights off; maintaining a balanced diet instead of being selective or picky about food; regularly performing eye exercises; taking eye breaks every 30 min when using electronic devices (e.g., looking into the distance, closing eyes); using a desk lamp when doing homework at night; attending regular hospital checkups for vision screening. Meanwhile, the experimental group's students demonstrated higher total scores for eye health behaviors compared to the control group (*P* < 0.05) (Table 4).

**Table 4:** Comparison of the practice of eye health behaviors between the two groups (points) [*M* (*p*25, *p*75)].

Practice	Experimental group (*n* = 461)	Control group (*n* = 443)	*Z*	*P*
1. Read, write or use electronic products while lying down with one's face down	3.00 (3.00, 3.00)	3.00 (3.00, 3.00)	−5.286	<0.001
2. Read, write or use electronic products while lying down	3.00 (3.00, 3.00)	3.00 (3.00, 3.00)	−5.102	<0.001
3. Read, write or use electronic products in a moving car	3.00 (3.00, 3.00)	3.00 (3.00, 3.00)	−6.648	<0.001
4. Read, write or use electronic products while walking	3.00 (3.00, 3.00)	3.00 (3.00, 3.00)	−5.491	<0.001
5. Read, write or use electronic products under direct sunlight or strong light	3.00 (3.00, 3.00)	3.00 (3.00, 3.00)	−3.703	<0.001
6. Read, write or use electronic products in dimly lit places	3.00 (3.00, 3.00)	3.00 (3.00, 3.00)	−4.715	<0.001
7. Watch TV at night with lights off	3.00 (3.00, 3.00)	3.00 (3.00, 3.00)	−7.332	<0.001
8. Have a picky diet	3.00 (3.00, 3.00)	3.00 (3.00, 3.00)	−4.779	<0.001
9. Adhere to eye exercises	3.00 (3.00, 3.00)	3.00 (3.00, 3.00)	−3.845	<0.001
10. Rest eyes after 30 min for continuous use (such as looking into the distance, closing eyes, etc.)	3.00 (3.00, 3.00)	2.00 (3.00, 3.00)	−4.428	<0.001
11. Do the homework with a desk lamp at night	3.00 (3.00, 3.00)	2.00 (3.00, 3.00)	−5.774	<0.001
12. Go to the hospital regularly for regular vision checkups	2.00 (2.00, 2.00)	2.00 (2.00, 2.00)	−2.216	0.027
Total score for the practice of eye health	35.00 (34.00, 35.00)	33.00 (32.00, 34.00)	−14.425	<0.001

### Comparison of satisfaction with eye health education between the two groups

One month after the intervention, the experimental group's students expressed higher satisfaction with both the form and content of the eye health education compared to the control group (*P* < 0.05) (Table 5).

**Table 5:** Comparison of the satisfaction with the eye health education between the two groups (points) [*M* (*p*25, *p*75)].

Satisfaction	Experimental group (*n* = 461)	Control group (*n* = 443)	*Z*	*P*
1. Is this eye health education timely?	5.00 (5.00, 5.00)	4.00 (5.00, 5.00)	−15.259	<0.001
2. Is the content of this eye health education helpful?	5.00 (5.00, 5.00)	4.00 (5.00, 5.00)	−12.280	<0.001
3. Are you satisfied with the interactions in this eye health education?	5.00 (5.00, 5.00)	4.00 (5.00, 5.00)	−11.472	<0.001
4. Are you satisfied with the form of this eye health education?	5.00 (5.00, 5.00)	4.00 (5.00, 5.00)	−9.451	<0.001
5. Is this eye health education novel or interesting?	5.00 (5.00, 5.00)	4.00 (5.00, 5.00)	−9.259	<0.001
Total score for the satisfaction with the eye health education	25.00 (24.00, 25.00)	22.00 (21.00, 24.00)	−20.504	<0.001

## Discussion

Identifying children at risk of developing myopia, often referred to as pre-myopic children, and implementing targeted prevention strategies, can drastically mitigate the impact of myopia on both individual well-being and societal health (11). Addressing myopia among children and adolescents necessitates a comprehensive and structured approach, emphasizing the importance of policy formulation, enhanced monitoring, standardized prevention protocols, technological advancements, and the establishment of demonstration zones for effective myopia prevention and control (12). Consequently, this study aimed to analyze the application value and feasibility of the precise eye health education model in the prevention and control of myopia among adolescents. The study's high effective response rate and full questionnaire recovery rate for both the KAP questionnaire and the satisfaction survey reinforce the methodological robustness of the study and its reliability in generating meaningful comparisons between groups.

The results demonstrated that the precision eye health education model, which incorporated a multifaceted approach—including the establishment of a health education team, the integration of an eye health intelligent management cloud platform, and the implementation of targeted health education content—was highly effective in enhancing students' KAP related to eye health. Specifically, students in the experimental group exhibited significantly higher total scores in eye health knowledge, attitudes, and behaviors compared to those in the control group. These findings underscore the value of structured KAP surveys as an essential assessment tool for analyzing individuals' perceptions, beliefs, and actions concerning myopia prevention and management (13). By leveraging KAP methodologies, researchers can gain deeper insights into the factors influencing the adoption of preventive behaviors and the effectiveness of management strategies for myopia control (14).

The experimental group demonstrated a stronger understanding of eye health, particularly regarding the importance of vision and its relationship with factors such as excessive eye use, reading and writing habits, and diet. They also exhibited greater awareness of the necessity for regular vision examinations and timely treatment for vision loss. In terms of attitudes toward eye health, the experimental group showed a more positive perspective on vision protection, displaying a stronger willingness to engage in physical exercise, improve poor dietary habits, and actively promote myopia prevention knowledge. Additionally, their eye health practices were notably better, as they were more likely to avoid reading, writing, or using electronic devices in unhealthy environments such as lying down, in a moving car, or in dimly lit areas. They also demonstrated higher adherence to eye exercises and were more consistent in resting their eyes after prolonged use. These improvements in knowledge, attitudes, and behaviors among the experimental group were likely to contribute to a reduction in the incidence of myopia within this population. Furthermore, the high level of satisfaction expressed by students regarding the eye health education further substantiates the effectiveness of the precision eye health education model. The satisfaction questionnaire, which assessed both the format and content of the educational intervention, indicated that students generally found the program informative, practical, and beneficial.

It is noteworthy that Babak Pezeshki's study, which applied the Health Belief Model (HBM) to enhance eye care performance among diabetics (15), aligns with the core principle of this research—leveraging the HBM to promote healthy behavioral changes. However, the two studies differ in focus: Pezeshki's study centers on eye health management among diabetics, while this study targets myopia prevention among adolescents, highlighting the broad applicability of the HBM across diverse health domains and populations.

Additionally, a school-family health education project utilizing the WeChat platform demonstrated a slight decline in the cumulative incidence of myopia over 2 years (16). This project underscores the crucial role of schools in myopia prevention and control, a perspective shared by this research. However, this study extends beyond school-based education by implementing a more refined eye health education model, which integrates personalized content customization and direct intervention in students' eye-use behaviors. This distinction may result in differences in long-term tracking and the sustainability of intervention effects. Similarly, Nan Jiang et al. explored parents' behavioral intentions regarding myopia prevention in preschool children by integrating the Health Belief Model with the Theory of Planned Behavior (17). Although their integrated model offers a comprehensive theoretical framework, this study emphasizes direct educational interventions targeted at adolescents. By assessing students' specific eye-use habits, the precision eye health education model delivers personalized educational content and intervention strategies tailored to individual needs.

In conclusion, this study demonstrates that the precision eye health education model represents a promising approach for myopia prevention and control in adolescents. Through its comprehensive, targeted, and personalized interventions, the model significantly enhances students’ eye health knowledge, attitudes, and behavioral practices. The research was conducted on a large, homogeneous population with similar baseline characteristics, reducing potential biases from individual differences and thereby enhancing the generalizability of the findings. These results contribute to the growing body of evidence supporting structured educational models in myopia prevention. Schools, parents, and society should consider adopting this model to promote eye health among children and adolescents. However, certain limitations of this study should be acknowledged. The reliance on self-reported survey questionnaires introduces a risk of information bias, as responses may be influenced by subjectivity and individual interpretation. Additionally, variations in comprehension abilities among respondents may affect the consistency of questionnaire responses. To mitigate these concerns, this study referenced existing validated surveys and incorporated expert revisions to enhance the accuracy and reliability of the questionnaires. For future research, further refinements to the intervention measures of the precision eye health education model are recommended. Developing more tailored educational content and intervention strategies for adolescents based on age and myopia risk level could enhance effectiveness. Additionally, extending the research period to track the long-term impact of the model will provide insights into its sustainability and stability in preventing myopia progression among adolescents.

## Data Availability

The original contributions presented in the study are included in the article/Supplementary Material, further inquiries can be directed to the corresponding author.
